# Correction: Oral glucose tolerance test clearance in type 2 diabetes patients who underwent remission following intense lifestyle modification: A quasi-experimental study

**DOI:** 10.1371/journal.pone.0315024

**Published:** 2024-12-02

**Authors:** Pramod Tripathi, Nidhi Kadam, Diptika Tiwari, Anagha Vyawahare, Baby Sharma, Thejas Kathrikolly, Maheshkumar Kuppusamy, Venugopal Vijayakumar

In the Abstract, there is an error in the seventh sentence of the paragraph. The correct sentence is: Pre-intervention use of glucose-lowering drugs showed no association with OGTT clearance (p>0.1).
