# Supplementary material for: Oral glucose tolerance test clearance in type 2 diabetes patients who underwent remission following intense lifestyle modification: A quasi-experimental study
Source: PLoS One. 2024 May 3;19(5):e0302777. doi: 10.1371/journal.pone.0302777 (PMC11068193; doi:10.1371/journal.pone.0302777)
Supplement: Attachment — Submitted filename: Response to Reviewers.docx [file pone.0302777.s002.docx]

**RESPONSE TO REVIEWERS**

We sincerely thank the editor and the reviewers for their valuable and insightful comments; they helped improve the quality of our manuscript. Please find below the detailed response to the reviewers’ comments for your kind perusal and necessary consideration.

**Additional Editor Comments:**

This study addresses a crucial aspect of diabetes management — the ability to achieve normal glycaemic profiles post-remission through lifestyle interventions. This is a significant area of research with potential implications for diabetes care. Additionally, the intervention includes various elements like diet, exercise, psychological support, and medical management, which collectively contribute to a holistic treatment approach. Furthermore, the authors have collected a wide range of data points, including HbA1c levels, insulin resistance, β-cell function, and anthropometric measures, providing a robust basis for analysis. Despite all these pros, this manuscript could be improved by considering the following.

**Comment 1**. The study does not isolate the effects of individual components of the lifestyle intervention, which could be insightful for understanding which elements are most effective. Discussing this could also improve the manuscript.

**Response: Thank you for your insightful comment. Due to the holistic nature of the intervention, we were unable to identify the isolated effect of individual components (Diet, Exercise, Psychological and Medical interventions) on T2D remission. We have added the same to the limitation section of the revised manuscript as below-**

**“Additionally, due to the holistic nature of the intervention with many factors at play, we could not study the isolated effect of any one component. Despite being a limitation, our research highlights the significant effectiveness of the lifestyle management program in achieving a better outcome in T2D management compared to a single intervention. The importance of holistic lifestyle intervention has been widely reported in several other studies, which highlights the importance of our findings in contributing to the body of knowledge in T2D management.”**

**Comment 2.** While the authors have reported statistical significance, the effect sizes of the interventions could be more explicitly stated to understand the magnitude of the changes observed.

**Response: Thank you for your feedback on this. As per your suggestion, we have now incorporated pre-post values in Tables nos. 1 and 2 to highlight the effect size and the magnitude of the changes observed.**

**Comment 3.** The introduction could more explicitly state the study’s hypotheses or research questions.

**Response: Thank you for your valuable suggestion. As suggested, we have revised the introduction to clearly state our hypothesis and research question as below-**

**“The current study aimed to investigate the association between OGTT clearance and T2D remission in patients who achieved remission after attending an online one-year lifestyle intervention program. The study hypothesis was that T2D-diagnosed patients in remission post-lifestyle intervention may be able to attain a normal glycemic profile through OGTT clearance, in addition to lowering HbA1c levels and improving insulin-related factors (HOMA-IR and HOMA-β).”**

**Comment 4.** The manuscript should address any ethical considerations, particularly given the intervention's comprehensive nature and the potential impact on patients' lifestyles.

**Response: As per the editor’s suggestions we have mentioned the implications of our interventions in the patients considering the ethical aspects, in the ‘Intervention section’ of the Methods as below-**

**“The ethical framework of our intervention emphasized the significance of individualized dietary modifications, customized exercise schedules, yoga, and stress management techniques, while simultaneously acknowledging the cultural nuances associated with dietary preferences and exercise choices. Our approach offered participants the flexibility to select from a range of options while still adhering to the primary objective of the intervention.”**

**Comment 5**. There may be an element of selection bias, as patients who opt into such a program might be more motivated or have different characteristics than the general diabetic population. This should be discussed.

**Response: We acknowledge the element of selection bias, as individuals opting to participate in our lifestyle management program may exhibit higher motivation levels or possess different characteristics compared to the general diabetic population. We are thankful to the reviewer for prompting this discussion, which enhances the transparency and integrity of our research. Since this is one of the limitations of our study we have discussed it in the Discussion section as below-**

**“Another limitation is the selection bias due to the voluntary nature of participation in our lifestyle management program. Those who choose to participate may be more motivated or have unique traits when compared to the broader diabetes community. This self-selection could impact the external validity of our findings. Although the results of our lifestyle intervention program may not generalize to the broader population with diabetes, this does not diminish the importance of our findings within a specific group of participants.”**

**Reviewer Comments**

**Reviewer #1:  I thank the authors for this relevant and informative study. However, the authors have overlooked some key issues as noted below.**

**Comment 1.** The authors report that their study is a quasi-experimental pre-post-study design, however, some key data is not clearly depicted. The authors report baseline, post-intervention and post OGTT data for HOMA-IR and HOMA-B (table 1 and Figure 2), however given they report 3 specific outcome groups (cleared, IGT and not cleared) the baseline and post intervention data should also be grouped as such. There is quite a wide range in the measurements for both HOMA-IR and HOMA-B and it would be easier to discuss the observations noted if the data is grouped as such.
The authors also use post-intervention in table 2 is confusing. Do they mean post the 1 year intervention or post the 3 months of glycaemic-ladder’ training? The table heading should be more clear.

**Response: Thank you for your comment. As per the suggestions we have revised and reported the baseline and post-intervention parameters in three groups based on OGTT status. We have also modified the tables and changed the headings. We have replaced Table 2 and Fig.2 with two new tables (Table 1 and 2) showing the comparison between and within the groups. To clarify, the three-month glycemic training is part of the one-year intervention for all those who are eligible. We have clarified this in the Methods section as below-**

**“As a part of the one-year lifestyle intervention program, upon achieving remission, participants were asked to voluntarily enrol for a three-month ‘glycemic-ladder’ training where they were gradually introduced to food items that were restricted during the intervention.”**

**Comment 2.** The authors also report P values showing significance differences between the groups, however, these are only mentioned in the text and broad terms. The P values are not depicted in any of the figures, and the figures are also lacking in the number of participants for each observation. If the authors were also able to group the baseline and post-intervention data into the three observations noted they would be able to compare differences in these parameters that could predict the 3 observations in the OGTT.

**Response: Thank you for the insightful comment on the data presentation. As per the suggestions, we have made the necessary changes and reported the exact p values and the participant numbers in the text as well as figures. We have also performed the analysis and compared them between the three groups based on OGTT results for all the parameters.**

**Comment 3.** Although the authors do report that the not-cleared group is small (n = 18), the results in this group warrant more exploration and discussion. In this group they observed improvements in HOMA-IR post-intervention however there was a decline in beta cell function as noted by HOMA-B measurements. In a previous diet intervention study of much lower duration (A Plant-Based Dietary Intervention Improves Beta-Cell Function and Insulin Resistance in Overweight Adults: A 16-week Randomized Clinical Trial, doi: 10.3390/nu10020189), improvement in beta-cell function was associated with improvements in HOMA-IR as is to be expected. The authors should discuss their unique results in more detail and offer plausible explanations for their observations.

**Response: Thank you for your comment. We recognise the limited sample size of the "not cleared" group (n = 18) as well as the intriguing findings that need additional investigation. While there were improvements in HOMA-IR following intervention, there was a concurrent drop in beta-cell function as measured by HOMA-β. This is in contrast to the previously found connection between enhanced beta-cell activity and decreased insulin resistance in shorter-term research (doi:** [**https://doi.org/10.3390/nu10020189**](https://doi.org/10.3390/nu10020189)**). While our findings may appear to be exceptional, the literature does include examples of lifestyle treatments that do not uniformly improve both HOMA-IR and HOMA-B levels. Several studies have found individual responses to be variable, emphasising the variety in metabolic outcomes.** **As suggested, we have incorporated the possible explanation for this** **in the discussion section as below-**

**“Despite low numbers, a substantial reduction in insulin resistance (HOMA-IR) was observed post-intervention in the Not-cleared group, yet the results remained suboptimal (HOMA-IR=2.8); concurrently there was a decrease in HOMA-β, contradicting Kahleova et al.’s 2019 [31] reports of improved beta-cell function and fasting insulin sensitivity in a 16-week dietary intervention for overweight individuals without diabetes history. The intricate interplay of genetic and age-related factors, baseline metabolic status, and diabetes duration may contribute to the observed variations [13,32]. The small sample size poses a challenge in drawing definitive conclusions, due to the inherent variability in our study population for the Not-cleared group, emphasizing the need for cautious interpretation. Future research with a larger sample size could delve into the specific mechanisms underlying the observed changes, concerning HOMA-IR and HOMA-β.”**

**Reviewer #2: General comment: You have a nice study if the following are included in your revision**

Title: Okay

Abstract:

-Does not highlight type of lifestyle modification intervention that was offered

**Response: As per the suggestion of the reviewer we have mentioned the type of lifestyle intervention offered to participants in the abstract as below-**

**“Four hundred participants who achieved remission during a one-year online structured lifestyle modification program, which included a plant-based diet, physical activity, psychological support, and medical management (between January 2021 and June 2022), and appeared for the OGTT were included in the study.”**

Introduction:

-what was the problem here?

-What was you testing? Effectiveness of an intervention or suitability of OGTT in relation to HbA1c? state clearly

**Response: Thank you for the valuable input. As suggested, we have revised the Introduction and added a clear statement of hypothesis along with clarity to what we aimed to study.**

Patient Enrolment:

-Why not call them participants?

**Response: Thank you for your suggestion. We have replaced the word patient with participants in all relevant places.**

-Do you think the nature of your sampling plan could have influenced your conclusion?

**Response: Thank you for your insightful comment. We agree that our sampling approach has limitations and we have discussed this in detail in the limitation section of our manuscript as below-**

**“Another limitation is the selection bias due to the voluntary nature of participation in our lifestyle management program. Those who choose to participate may be more motivated or have unique traits when compared to the broader diabetes community. This self-selection could impact the external validity of our findings. Although the results of our lifestyle intervention program may not generalize to the broader population with diabetes, this does not diminish the importance of our findings within a specific group of participants.”**

**However, we want to highlight that our primary aim was to investigate the possibility of achieving normal glucose levels in T2D-diagnosed participants under remission. Despite the limitations, our study provides valuable insights into the relationship between OGTT clearance and remission and adds new data to the existing body of knowledge. In future studies planned, we will make sure to adopt randomization in our sampling.**

-do you think the potential participants who achieved remission differed from those who didn’t? and what about those who were not included in the final analysis? Would the results and hence conclusion been different if all who achieved remission had participated? Why?

**Response: Thank you for your insightful comment. Based on our analysis for another paper on diabetes remission, we found significant differences between those who achieved remission vs those who did not especially in terms of weight loss and diabetes duration- those with weight loss of more than 15 kg and diabetes duration less than 6 years showed higher remission rates (unpublished data).**

**In the present study, since our focus was on individuals experiencing remission, the inclusion criteria mandated the enrollment of participants under remission. To answer your second query on those who enrolled but were not included in the final analysis, we conducted a preliminary analysis of Biochemical and Anthropometric parameters on the non-participant group (N=761) and notably, the results for non-participants also showed significant improvements for all the study parameters (Table given below) indicating a lesser chance of affecting our study outcome.**

| **Parameters** | **Drop out Participant under remission (N=761)** | | P values |
| --- | --- | --- | --- |
|  | Pre-Intervention | Post Intervention |  |
| **Weight (kg)** | 72 (65-81.3) | 67.5 (60-75) | <0.001 |
| **BMI (kg/m^2^)** | 25.8 (23.8-28.7) | 23.8 (22.5-26.4) | <0.001 |
| **HbA1c (mmol/mol)** | 51.9 (45.3- 61.7) | 43.1 (38.7- 47.5) | <0.001 |
| **Fasting Insulin (pmol/L)** | 49.8 (34.5-76.9) | 41.4 (27.9 - 64.5) | <0.001 |
| **Fasting Blood Sugar (mmol/L)** | 6.6 (5.7 – 7.7) | 6.1(5.4 - 6.9) | <0.001 |
| **Post Prandial Blood Sugar (mmol/L)** | 7.6 (6.1 – 9.8) | 7.0 (5.8 -8.8) | <0.001 |
| **HOMA-IR** | 2.6 (1.6 – 4.1) | 1.9 (1.2 - 2.9) | <0.001 |
| **HOMA-β** | 51.6 (31.1 – 88.7) | 52.2 (32.1 -88.9) | 0.314 |

**We do acknowledge that a study including all participants in remission would have strengthened the overall validity and applicability of our findings. However, we had to consider the voluntary aspect of taking part in the current study.**

Inclusion Criteria:

- did you account for potential contamination from alternative glucose-lowering therapies such as herbs which could have not been part of the intervention?

**Response: Based on the eligibility criteria we considered only those participants who were off all glucose-lowering medicines including alternative medicine. We have added the same in the inclusion criteria for clarity as below-**

**“The primary inclusion criterion was remission defined as ‘HbA1c less than 48 mmol/mol without any glucose-lowering medicines (including alternative medicine) for at least 3 months…”**

Intervention:

-What was the intervention? One year lifestyle modification versus 3-month glycaemic training.

**Response: The intervention was an online one-year lifestyle modification programme that comprised dietary, exercise, psychological and medical management. The 3-month glycemic training was a part of the intervention for all those participants who underwent remission during the program. All those who achieved remission were invited to undergo the 3-month glycemic training during the one year they were with us. We have stated this more clearly in the Intervention Section of our manuscript as below-**

**“As a part of the one-year lifestyle intervention program, upon achieving remission, participants were asked to voluntarily enrol for a three-month ‘glycemic-ladder’ training where they were gradually introduced to food items that were restricted during the intervention.”**

-why were all participants kept under the same calories target at a given stage? Why was the target not individualized depending on personal characteristics? Do you think that could have influenced your results?

**Response:** **Thank you for your comment. We apologise if this was not mentioned clearly in our text earlier but the one-year lifestyle intervention was personalized and customized as per the participants'** **BMI. For those with BMI in the normal range, juice and water fasting were restricted. For those with higher BMI, higher calorie restriction through juice and water fasting was advised. We have added the same to the Intervention section of the manuscript as below-**

**“Dietary modification was individualised based on the BMI and associated medical conditions of the participants”**

**“In the second month, calorie intake was further reduced (400-500 kcal/day on the day of fasting) through intermittent fasting and juice fasting to aid in faster weight loss, depending on the BMI of the participant; those with normal BMI did not undergo juice and water fasting. Once the target BMI (23-25 kg/m2) was achieved, the calorie intake was gradually increased to 1800-2000 kcal to promote muscle building (with the aid of exercises).”**

-how did you take into account non-exercise physical activities such as those related to work?

**Response: Thank you for your valuable observation. Our study addressed non-exercise physical activities such as those related to work by collecting detailed occupation-related data during the enrollment process. The study revealed that a notable percentage (64.4%) of the population had a sedentary lifestyle, mainly in office settings. Overall homogeneous work-related physical activity pattern was observed among the population.**

-how was compliance to intervention ensured and assessed?

**Response:** **During the intervention, adherence was assessed monthly, for a total of 12 assessments throughout the programme. The rigorous monthly adherence checks guaranteed that participants' engagement with the intervention components was thoroughly and continuously assessed. We have added the same in the Intervention section as below-**

**“Adherence to the overall programme was monitored through 12 structured questionnaires administered monthly through our mobile application. This ensured the engagement of participants and provided direct feedback to all respective experts to take necessary action to ensure adherence to the protocol.”**

-Psychological support was part of the intervention but you say nothing in the introduction

**Response: As highlighted by the reviewer we have mentioned the importance of Psychological support in the Introduction section as below-**

**“The American Diabetes Association (ADA) has recommended an assessment of psychosocial and emotional health concerns in patients with diabetes, making it an integral part of holistic diabetes care in addition to conventional lifestyle changes”**

-why interventions appear here but nothing in results?

**Response: Thank you for drawing our attention to the fact that interventions are mentioned in the methodology's Intervention section but not in the Results section. As per the suggestion, we have included a brief overview of the intervention in the Results section as below-**

**“The results presented here reflect the outcomes observed after a one-year lifestyle intervention program for diabetes management. The participants underwent a comprehensive one-year intervention that included dietary, exercise, psychological and medical management. Four hundred participants who fulfilled the remission criteria, completed the three-month ‘glycemic ladder’ training, and underwent the oral glucose tolerance test (OGTT) during the one-year intervention were included in the final analysis.”**

-Participants had individualized exercise sessions, how are they comparable to each other?

**Response:** **We appreciate the reviewer's query regarding the comparability of individualized exercise sessions in our study. We used a systematic framework known as the 3-2-1 pattern while participants were doing individual workout sessions. This pattern emphasized three key components: strength, stamina, and flexibility. All participants had the freedom to select from a variety of possibilities within each category based on their fitness levels, endurance and health considerations. While the specific exercises may have varied, the underlying emphasis on strength, stamina, and flexibility ensured a commonality in the core objectives of the exercise sessions. We believe that a mix of individualization and a standardized framework contributed to a more tailored and yet comparable intervention across participants, allowing for valuable insights into the impact of exercise on the outcomes of interest. We have added the same in the intervention section as below-**

**“The exercise intervention included exercises to promote blood circulation and muscle activation (warm-up, Surya Namaskar, super brain yoga, palm planks), with a major focus on improving strength, flexibility, and stamina by adopting the 3-2-1 pattern per week (3 hrs. of strength, 2 hrs of flexibility and 1 hrs. of stamina exercises). A customised exercise plan (e.g., swimming, running, cycling, yoga) was suggested to the participants based on their age and preference to create a consistent level of fitness by the end of the program.”**

Results:

-What was the composition of weight lost (water, fat or lean mass)? How was is measured? Do you think it could have influenced the results?

# **Response:** **We appreciate the reviewer's intelligent inquiry regarding our study’s weight loss composition. Due to the nature of our online intervention programme, which did not entail in-person consults with participants, we were unable to directly quantify the composition of weight loss, such as water, fat, or lean mass.**

**While we acknowledge that such measurements could have provided a more detailed understanding of the mechanism of weight loss, our study primarily focused on the overall impact of the intervention on remission in T2D-diagnosed patients. We recognise the importance of investigating the composition of weight loss to gain a better understanding of the physiological changes associated with the intervention and will plan future research accordingly.**

-Pre-intervention BMI range (21.4-32.4) indicates underweight patients were included in the weight-losing intervention. The post-intervention BMI range (17.3-28.2) indicated that some could have gone to severer underweight. What is your intervention ethical?

**Response: The reviewer's insightful observation regarding the BMI ranges in our study is greatly appreciated. Although our pre-intervention BMI range did encompass underweight patients, and the post-intervention BMI range suggests potential weight reduction, it is essential to clarify that our intervention's primary objective was to achieve T2D remission. The dietary intervention was initially centered on a plant-based diet, with modifications tailored to each participant's BMI and associated medical conditions. While weight reduction is a vital aspect of the overall outcome, our primary focus was on addressing the underlying metabolic factors associated with diabetes. The inclusion of a diverse BMI range aims to capture the spectrum of individuals affected by T2D. We have emphasized this perspective in the manuscript to ensure a comprehensive understanding of our intervention's goals and outcomes.**

**The hypothesis put forth by Dr. Roy Taylor, known as the "Personal Fat Threshold" (PFT) hypothesis, posits that each individual has a specific limit to the amount of fat they can accumulate, and exceeding this threshold leads to the development of type 2 diabetes (T2D). It is important to note that this PFT is separate from an individual's Body Mass Index (BMI) and that weight loss can bring a person below their level of susceptibility, allowing them to regain normal glucose control. The ReTUNE trial provides evidence supporting this hypothesis, demonstrating that weight loss can improve HbA1c levels in individuals with normal or near-normal BMI (Taylor, R., & Holman, R. R. (2015). <https://doi.org/10.1042/CS20140553>, (Clinical science (London, England: 1979), 137(16), 1333–1346. <https://doi.org/10.1042/CS20230586>).**

**To address ethical concerns, it is essential to emphasize that the safety of participants was always a top priority throughout the intervention. Strict monitoring of protocols was conducted to closely track changes in BMI, and individualized interventions were tailored to ensure that weight loss occurred within safe and controlled parameters. During the intervention period, no adverse events were reported in individuals with normal BMI who underwent weight loss.**

-your title and introduction do not indicate if you were going to study the Homeostatic model

assessment of Insulin resistance (HOMA-IR); HOMA-β: Homeostatic model assessment of

β-cell function

**Response: Thank you for the suggestion. We have added the same to the Introduction section as below-**

**“Long-term remission in T2D-diagnosed patients is most predicted by sustained weight loss, lower duration of diabetes, and reduced insulin resistance, in addition to improved pancreatic function [11,12] Multiple studies suggest that long-term maintenance of remission and prevention of relapse in T2D is mostly achieved by long-term adherence to lifestyle therapy and weight loss, provided β-cell dysfunction is not irreparable”.**

Discussion:

-revisit the statement “To our knowledge, this is the first study to assess T2D remission in terms of achieving a normal glucose profile post-lifestyle intervention”

**Response: Thank you for your comment. Based on the suggestion, we have reframed the sentence to “As far as our knowledge extends, no previous research has investigated the remission of Type 2 diabetes with a focus on attaining a normal glucose profile, as assessed through an oral glucose tolerance test (OGTT) following a lifestyle intervention.”**

**We would like to highlight that although OGTT is used as a diagnostic tool for dysglycemic status and as a measurement criterion for remission (**<https://doi.org/10.2337/dc22-S002>**; Kuo et al. 2021** <https://doi.org/10.4239%2Fwjd.v12.i6.786>**; Thewjitcharoen et al. 2019,** <https://rdcu.be/dtYDz>), **our literature review showed no existing studies on the association of achieving a normal glucose profile after one year of lifestyle intervention in T2D patients under remission. We consider this as one of the strengths of our study.**

Conclusion

-Conclude on insulin resistance and B-cell function

**Response: As suggested by the esteemed reviewer we have included the findings on Insulin Resistance and Beta Cell Function in the Conclusion section of the manuscript as below-**

**“Additionally, improvements in insulin resistance markers suggest their role in increasing the chances of achieving normal glycemic control in patients with T2D.”**
