# Supplementary material for: Oral glucose tolerance test clearance in type 2 diabetes patients who underwent remission following intense lifestyle modification: A quasi-experimental study
Source: PLoS One. 2024 May 3;19(5):e0302777. doi: 10.1371/journal.pone.0302777 (PMC11068193; doi:10.1371/journal.pone.0302777)
Supplement: Attachment — Submitted filename: Response to Reviewers.docx [file pone.0302777.s003.docx]

**RESPONSE TO REVIEWERS**

We sincerely thank the editor and the reviewers for their valuable and insightful comments; they helped improve the quality of our manuscript. Please find below the detailed response to the reviewers’ comments for your kind perusal and necessary consideration.

**Additional Editor Comments:**

Please address thoroughly the comments raised by one of the reviewers. Make sure you address them clearly as recommended.

**Reviewer Comments**

**Reviewer #1:**

**Comment 1.** In the abstract as well as in the introduction and discussion the authors indicate that they they want to assess the association between OGTT clearance and diabetes remission. However, no statistical analyses were done to assess for association.

In the final paragraph the authors write "However, there is a lack of studies emphasizing the correlation between a normal glycemic profile in terms of OGTT clearance and T2D remission. The current study aimed to investigate the association between OGTT clearance and T2D remission in patients..." Neither association nor correlations were assessed in their results.

***Response:*** Thank you for this comment. We accept our error in stating that we “aimed to investigate *the association between OGTT clearance and T2D remission*.” Since all participants in the study were already in remission, our study aimed to assess the possibility of OGTT clearance in patients who successfully achieved remission after one year of lifestyle intervention and to explore the factors associated with OGTT status. We have revised the same in our manuscript in the abstract as well as the introduction section, as below-

Abstract:

*“The study investigates the factors associated with OGTT clearance in patients under type 2 diabetes remission.”*

Introduction:

“*However, there is a lack of studies exploring the possibility of achieving a normal glycemic profile in terms of OGTT clearance post-T2D remission. The current study aimed to investigate the factors associated with OGTT clearance in T2D patients who achieved remission after attending an online one-year lifestyle intervention program.”*

**Comment 2.** The methodology of the intervention is still unclear. The authors have responded that the glycemic ladder training occured in the course of the year of the lifestyle modification after which the OGTT was conducted. However how was this process influenced by the timetaken to attain remission? If a patient eg attained remission after 2 months VS 11 months, when were the glycemic ladder training and OGTT conducted for the two patients? what were the mean durations for remission for the three groups?

***Response:*** Thank you for your insightful comment. Remission was considered if the participant was able to maintain their HbA1c levels at less than 6.5% for at least 3 months after stopping all medications. Only 3 months after the complete cessation of medication, based on the HbA1c levels, the glycemic ladder training was initiated. Overall, the mean time taken to achieve remission before starting glycemic ladder training was 4.6 ± 1.9 months. The remission duration for the 3 groups was as follows: OGTT cleared group: 4.4 ± 2.0 months; IGT group: 4.7 ± 1.8 months; Not cleared group: 5.6 ± 2.5 months. Although the remission duration was the longest in the non-cleared group, we were unable to test for significance between groups due to the small sample size in the non-cleared group (n=18).

Most patients achieved remission within a one-year program. For those attaining remission towards the end of the program, glycemic ladder training support was extended through WhatsApp groups specially created for the study. The participants were allowed to submit their reports after the completion of the program. This was possible because of the personalized nature of the program. Therefore, if a participant attained remission early during the program, he started glycemic training earlier than someone who achieved remission later.

We have added the data to the results section of the manuscript as below-

“*Overall,* *the mean time taken to achieve remission before starting glycemic ladder training was 4.6 ± 1.9 months.”*

*“The mean time taken to achieve remission before starting glycemic ladder training for the 3 groups was: OGTT cleared group, 4.4 ± 2.0 months; IGT group, 4.7 ± 1.8 months; Not cleared group, 5.6 ± 2.5 months.”*

**Comment 3:** Results section is much improved. I would urge the authors to add a note at the bottom of table 2 explainig to the reader whether the more negative or positive the change for the parameters assessed reflects benefit.

Clinical characteristics such as mean age, gender etc of participants in the three groups is lacking and could shed light on the observations noted (doi: 10.2337/dc14-0874**)**

***Response:*** Thank you for encouraging feedback on the Results section. As per the suggestions, we have revised and added a note to Table 2 to clarify whether the more negative or positive change in parameters reflects the benefit to enhance the reader's understanding as below-

*“The percentage decrease in weight, BMI, HbA1c, Fasting Insulin, Fasting Blood Sugar, PP-Blood Sugar, and HOMA-IR along with a percentage increase in HOMA-β indicates a positive effect and benefit to the participants.”*

Additionally, to address the 2^nd^ comment, we have added a new Table 1 to the Results section with sociodemographic characteristics (age, gender distribution, education, occupation, and marital status) for the three groups. The original figures 1A and 1B highlight other parameters such as diabetes duration and diabetes medication status (OHA and Insulin) that have been shifted after Table 1 to maintain continuity. The revised statement and table in the Results section have been incorporated as follows:

*“The sociodemographic profiles of the participants in the three groups are presented in Table 1. All three groups had similar sociodemographic characteristics (P >0.1). Diabetes duration was significantly lower in the OGTT-cleared group than in the IGT group (p=0.046) (Fig 1A). Due to the low number of patients who did not clear the OGTT (N = 18), we could not statistically compare the outcome for this group. No significant difference in OGTT results was observed based on initial medication status (p>0.1) (Fig 1B).”*

***Table 1: Sociodemographic profile of participants in three outcome groups (N=400)***

| ***Parameters*** | | | ***OGTT Status*** | | | | | |
| --- | --- | --- | --- | --- | --- | --- | --- | --- |
|  |  |  | ***OGTT-cleared (N=207)*** | | ***IGT***  ***(N=175)*** | | ***Not-cleared***  ***(N=18)*** | |
| *Age (Years)* | | | *52.0±9.4* | | *51.1±10.0* | | *52.8±9.8* | |
| *Gender* | *Male* | *116 [56.0]* | | *107 [61.1]* | | *12 [66.6]* | |  |
|  | *Female* | *91 [44.0]* | | *68 [38.9]* | | *6 [33.4]* | |  |
| *Education* | *Graduate and below* | *109 [52.7]* | | *98[56.0]* | | *12 [66.7]* | |  |
|  | *Post-Graduate and above* | *98 [47.3]* | | *77[44.0]* | | *6 [33.3]* | |  |
| *Occupation* | *Employed* | *118 [57.0]* | | *113 [64.6]* | | *13 [70.6]* | |  |
|  | *Retired* | *28 [13.5]* | | *19 [10.8]* | | *3 [17.6]* | |  |
|  | *Homemaker* | *39 [18.8]* | | *32 [18.3]* | | *2 [11.8]* | |  |
|  | *Others ^a^* | *22 [10.6]* | | *11 [6.3]* | | *-* | |  |
| *Marital Status* | *Married* | *184 [88.8]* | | *156 [89.1]* | | *17 [94.1]* | |  |
|  | *Single ^b^* | *23 [11.2]* | | *19 [10.9]* | | *1 [5.9]* | |  |
| *Family History* | *Yes* | *160 [77.1]* | | *133 [76.0]* | | *14 [76.5]* | |  |
|  | *No* | *47 [22.9]* | | *42 [24.0]* | | *4 [23.5]* | |  |

*Data are presented as mean ± standard deviation or frequency [percentage]; ^a^ Student/Preferred not to disclose; ^b^ Never married/separated/widowed; No significant differences were observed between the groups by Chi-square test (p>0.1)*

Tables 1 and 2 are relabeled in Tables 2 and 3, respectively.

**Reviewer #2: All the raised comments have been thoroughly and sufficiently addressed. The authors have made all necessary changes asked for by reviewers.**

***Response:*** Thank you for your thorough review and constructive comments. We appreciate your valuable input that has undoubtedly improved the clarity and quality of our manuscript.
